# Gut Microbiome-Mediated Genetic and Epigenetic Alterations in Colorectal Cancer: Population-Specific Insights

**DOI:** 10.3390/biomedicines13092262

**Published:** 2025-09-14

**Authors:** Simona Turcu, Florin Grama, Maria Gazouli

**Affiliations:** 1 Department of Surgery, Carol Davila University of Medicine and Pharmacy, 050474 Bucharest, Romania; simona.manciu@umfcd.ro (S.T.); florin.grama@umfcd.ro (F.G.); 2 Fundeni Clinical Institute, 022328 Bucharest, Romania; 3 Coltea Clinical Hospital, Ion C. Brătianu Boulevard 1, 030167 Bucharest, Romania; 4 Laboratory of Biology, Department of Basic Medical Sciences, Medical School, National and Kapodistrian University of Athens, 11527 Athens, Greece

**Keywords:** colorectal cancer, miRNAs, microbiome, *Fusobacterium nucleatum*, dysbiosis

## Abstract

Colorectal cancer (CRC) remains a major global challenge, with growing attention to its pathogenesis as mediated by the gut microbiome and epigenetic regulation. Despite therapeutic progress, clinical management remains difficult. CRC accounts for ~10% of cancers and is the second leading cause of cancer death worldwide. Romania bears a substantial burden, with many diagnoses at advanced stages. Etiology—Integrated Genetic, Environmental, and Microbial Determinants. Hereditary syndromes explain 10–15% of cases; most are sporadic, with hypermutated MSI/POLE (~15%), non-hypermutated chromosomal instability (~85%), and a CpG island methylator phenotype (~20%). GWAS implicate loci near SMAD7, TCF7L2, and CDH1; in Romania, SMAD7 rs4939827 associates with risk. Lifestyle exposures—high red/processed meat, low fiber, adiposity, alcohol, and smoking—shape susceptibility. Microbiome–Epigenome Interactions. Dysbiosis promotes carcinogenesis via genotoxins (e.g., colibactin), hydrogen sulfide, activation of NF-κB/STAT3, barrier disruption, and epigenetic remodeling of DNA methylation and microRNAs. *Fusobacterium nucleatum*, enterotoxigenic Bacteroides fragilis, and pks^+^ Escherichia coli exemplifies these links. Population-Specific Risk—Romania within Lifestyle–Microbiome Evidence. Incidence is rising, including early-onset disease. Romania lacks CRC-specific microbiome datasets. However, metabolic cohorts show loss of butyrate producers, enrichment of pathobionts, and SCFA imbalance—patterns that mirror European CRC cohorts—and exhibit regional heterogeneity. Beyond *Fusobacterium nucleatum*. Additional oncobacteria shape tumor biology. *Peptostreptococcus stomatis* activates integrin α6/β4→ERBB2–MAPK and can bypass targeted inhibitors, while *Parvimonas micra* enhances WNT/β-catenin programs and Th17-skewed immunity. Together, these data support a systems view in which microbial cues and host epigenetic control jointly drive CRC initiation, progression, metastasis, and treatment response.

## 1. Introduction: Reframing Colorectal Cancer Through the Gut Microbiome–Epigenome Axis

Colorectal cancer, encompassing both colon and rectal malignancies, is a growing public health concern. It ranks among the most prevalent cancers worldwide—after lung, breast (in women), and prostate—by incidence [1]. Despite advances in research and therapy, CRC remains challenging because of drug resistance, metastasis, and recurrence [2].

According to the World Health Organization, CRC accounts for ~10% of all cancer cases and is the second leading cause of cancer mortality. By 2040, the global burden is projected to exceed 3.2 million new cases and 1.6 million deaths annually—a ~66% increase in incidence compared with 2020 [3].

The picture is also concerning in Eastern Europe, where CRC mortality ranks among the highest in the region. In Romania, CRC accounts for roughly 20% of new cancer diagnoses in both sexes, and many cases are detected at advanced stages [4]. Although updated data are not yet available, recent estimates report a mortality rate of 14.9 per 100,000 and a hospitalization prevalence of 116.83 per 100,000. In 2020 alone, nearly 13,000 new cases were diagnosed, and more than 6000 deaths were attributed to CRC. The predominance of late-stage detection underscores persistent gaps in early diagnosis and timely intervention [5].

Early-onset colorectal cancer (EOCRC)—defined as CRC diagnosed before age 50—is increasing steeply, with projections of a >140% global rise by 2030 [6]. This shift suggests that environmental, dietary, and possibly microbial drivers are accelerating carcinogenesis in younger populations [7].

CRC is no longer viewed as a disease driven solely by genetic mutations. A growing body of research supports a paradigm in which tumorigenesis results from a triad of gene–environment–microbiota interactions [8]. Within this model, the gut microbiome acts not as a passive bystander but as an active modulator of the colonic microenvironment. Its metabolic output—ranging from pro-inflammatory molecules to epigenetically active metabolites—can influence host gene expression and immune signaling.

Epigenetic regulation, particularly through microRNAs (miRNAs), plays a pivotal role in this interaction, positioning miRNAs at the crossroads between epigenetic control and the gut microbiome. They are not only regulated by epigenetic mechanisms such as DNA methylation and histone modification but can also modulate epigenetic enzymes, creating reciprocal feedback loops that shape gene expression and cellular homeostasis. Adding further complexity, host–microbiome crosstalk influences these networks: gut microbes can alter host miRNA expression, while host-derived miRNAs can, in turn, regulate microbial gene activity. Disruption of this interconnected system contributes to cancer initiation and progression. We examine CRC through the microbiome–epigenome axis. We synthesize current evidence on how gut dysbiosis contributes to CRC via miRNA dysregulation and related epigenetic mechanisms, with a focus on Romania, a country facing a growing burden. We also consider how regional data on diet, genetics, and microbial patterns can inform prevention, diagnosis, and personalized treatment strategies.

The discussion is organized into four interconnected themes:

(1) Integrated determinants of CRC—the interplay of inherited susceptibility, lifestyle and environmental exposures, and the gut microbiome;
(2) Microbiome–epigenome mechanisms—how dysbiosis, via microRNA-centered and chromatin-mediated pathways, drives tumor initiation, progression, and treatment response;
(3) Population context for Romania—rising incidence and regional disparities interpreted alongside European lifestyle–microbiome evidence, with hypothesis-generating insights from Romanian metabolic cohorts;
(4) Broader oncobacterial contributions and translation—roles of taxa beyond *Fusobacterium nucleatum* and implications for biomarker development and precision oncology.

## 2. Colorectal Cancer Etiology: Integrated Genetic, Environmental, and Microbial Determinants

CRC is a multifactorial disease shaped by inherited susceptibility, environmental exposures, and endogenous biological perturbations. Within this framework, the gut microbiome functions as a pivotal internal environmental layer—modulating inflammation, metabolism, and epigenetic control—and thereby shaping how genetic risk and lifestyle exposures translate into tumorigenesis. CRC occurs either as hereditary syndromes caused by germline defects or, more commonly, as sporadic disease driven by the stepwise accumulation of somatic alterations shaped by environmental exposures.

Hereditary syndromes such as Lynch syndrome and familial adenomatous polyposis (FAP)—together ~10–15% of cases—have illuminated core pathways through germline defects in MLH1, MSH2, and APC. Most cancers are sporadic and arise through stepwise accumulation of somatic alterations, often molded by diet, inflammation, and other environmental inputs [9,10,11]. Molecularly, sporadic tumors segregate into recognizable patterns: a hypermutated subset (~15%) with mismatch-repair deficiency, microsatellite instability (MSI), or POLE mutations; a non-hypermutated majority (~85%) with chromosomal instability and recurrent mutations in APC, TP53, KRAS, and PIK3CA; a CpG island methylator phenotype (CIMP) in ~20% of cases; and a high prevalence of EMAST, which cuts across groups and associates with metastatic behavior. These features increasingly inform diagnosis, prognosis, and treatment selection beyond anatomic staging [12].

Genetic risk rarely acts in isolation. Genome-Wide Association Studies (GWAS) have identified common variants—such as loci near SMAD7, TCF7L2, and CDH1—that modulate how lifestyle exposures (alcohol, adiposity, diet, NSAID use) influence CRC susceptibility [13,14]. Population-specific data support this coupling: a Romanian case–control study linked SMAD7 rs4939827 with elevated risk, suggesting interactions between Westernized diets and local genetic architecture [15].

Lifestyle and environmental factors remain central levers of risk. High intake of processed and red meat, low dietary fiber, excess body weight, smoking, and alcohol use drive inflammatory signaling, oxidative stress, and imbalances in proliferation and apoptosis [16]. Country-specific exposure patterns shape local burden. In Romania, processed-meat consumption has increased; one study reported up to a sixfold rise in colon cancer risk among high consumers. Westernized diets and sedentary lifestyles likely contribute to rising incidence, including among younger adults [17].

Superimposed on these determinants is the gut microbiome. Dysbiosis—an imbalance in these microorganisms—can promote carcinogenesis through convergent mechanisms, including the production of genotoxins and metabolites (e.g., colibactin, hydrogen sulfide), activation of NF-κB/STAT3 signaling, disruption of epithelial barrier integrity, and epigenetic remodeling, such as altered DNA methylation and miRNA expression. Microbes most consistently linked to neoplasia illustrate how bacterial cues can hard-wire tumor-promoting programs in the colonic mucosa [18,19,20]. A detailed exposition of their mechanisms is provided in the subsequent sections, emphasizing miRNA-centered and epigenetic pathways through which microbial drivers shape CRC pathogenesis.

## 3. Microbiome–Epigenome Interactions in Colorectal Carcinogenesis: A MicroRNA-Centric Framework

The human microbiome encompasses a diverse community of microorganisms, including bacteria, archaea, fungi, and viruses that colonize various body sites. Predominantly bacterial, these microbes form symbiotic relationships with the host and influence key physiological processes by adapting to each specific niche [21]. In the colon, the mucosa engages in continuous cross-talk with this dense community. When community structure departs from eubiosis (dysbiosis), consequences extend beyond sustained inflammation. Imbalanced microbiota can reprogram epithelial gene regulation via microRNAs and chromatin-modifying enzymes, altering cell survival, invasiveness, immune evasion, and therapy responsiveness. miRNAs are central to this crosstalk. Gut microbes alter host miRNA programs through secreted metabolites and toxins, engagement of pattern-recognition pathways, and secondary effects on chromatin structure. Reciprocally, intestinal epithelial cells release exosomal miRNAs into the lumen, where resident bacteria can take them up and adjust microbial gene expression [22,23,24]. Within this bidirectional framework, certain taxa are repeatedly enriched in colorectal tumors and precursors—*F. nucleatum*, enterotoxigenic *Bacteroides fragilis* (ETBF), and genotoxic *Escherichia coli* [25,26,27,28]—while beneficial commensals such as *Faecalibacterium prausnitzii* and *Akkermansia muciniphila* are often depleted [29,30].

### 3.1. Microbial Taxa Affect miRNA Regulation in Colorectal Tissue

Among CRC-enriched taxa, *F. nucleatum* is consistently more abundant in adenomas and carcinomas than in matched normal mucosa and possesses virulence factors—FadA, Fap2, and FomA—that enable adhesion to and invasion of epithelial and endothelial cells [31,32,33,34]. One salient axis centers on miR-21, a colon cancer-associated miRNA. *F. nucleatum* upregulates miR-21, which inhibits RASA1, a RAS GTPase-activating protein that normally restrains the RAS pathway; removal of this control activates the RAS–MAPK cascade, thereby promoting cell proliferation and survival. This remodeling is intertwined with innate immunity: bacterial ligands engage the TLR4–MYD88 module in colonocytes, driving NF-κB activation and a pro-inflammatory transcriptional program that favors tumor growth, invasion, and metastasis [35]. Additional miRNA circuits expand *Fusobacterium*’s impact. Infection raises miR-31, which targets eIF4EBP1/2—repressors of cap-dependent translation—biasing protein synthesis toward tumorigenic outputs; high phosphorylated eIF4EBP1 has been linked to poor prognosis. The same miRNA downregulates STX12, a vesicular trafficking factor, impeding autophagosome–lysosome fusion and tilting cellular homeostasis toward tumor promotion [36].

*F. nucleatum* also contributes to therapy failure and dissemination. Through TLR4–MYD88 signaling it suppresses miR-18a* and miR-4802, two miRNAs that normally restrain ULK1 and ATG7; their loss increases autophagic flux and allows cancer cells to tolerate oxaliplatin and 5-fluorouracil. Restoring these miRNAs or interrupting upstream signaling reverses resistance [37].

Metastatic spread is amplified via a chemokine switch: the bacterium lowers miR-1322, de-repressing CCL20—one of the most strongly induced chemokines in tumors with high fusobacterial burden. Elevated CCL20 enhances recruitment of CCR6^+^ immune cells and supports lung colonization in experimental models; CCL20 knockdown or miR-1322 mimicry curtails *Fusobacterium*-driven metastatic outgrowth [38].

ETBF further illustrates how junctional injury couples to RNA-based control. ETBF carries the *bft* gene encoding Bacteroides fragilis toxin (BFT), which cleaves E-cadherin, disrupts tight junctions, and increases barrier permeability—perturbations believed to facilitate the normal–adenoma–carcinoma sequence. Beyond barrier damage, ETBF upregulates the long non-coding RNA BFAL1, which acts as a competitive “sponge” for miR-155-5p and miR-200a-3p. By relieving repression of RHEB, BFAL1 activates mTORC1, a nutrient-sensing growth complex that promotes protein synthesis and suppresses autophagy. In CRC cells, inhibitors of miR-155-5p and miR-200a-3p raise RHEB mRNA and protein, whereas miRNA mimics reduce them, consistent with direct 3′-UTR targeting; elevated RHEB correlates with poor differentiation, higher Dukes stage, and nodal metastasis, linking this circuit to aggressive biology [39,40].

Adherent–invasive *E. coli* (AIEC) mediates the link between inflammation and autophagy regulation through miRNAs. In human T84 epithelial cells and mouse enterocytes, infection with the AIEC strain LF82 activates NF-κB and induces miR-30c and miR-130a. These miRNAs directly downregulate ATG5 and ATG16L1—proteins that form an essential complex for autophagosome formation—thereby suppressing autophagy, increasing intracellular bacterial burden, and amplifying IL-8–driven inflammation. Gain- and loss-of-function studies confirm 3′-UTR-dependent regulation of ATG5/ATG16L1 by miR-30c/miR-130a. Pharmacologic NF-κB inhibition (PDTC) blocks miRNA induction, rescues autophagy genes, and dampens the inflammatory cascade [25].

These circuits show how *F. nucleatum*, ETBF, and AIEC translate adhesion, toxin delivery, and innate sensing into post-transcriptional reprogramming of the host. Yet microbial control does not stop at miRNAs. The same exposures reshape the epithelial epigenome through DNA methylation and histone modification, with short-chain fatty acids (SCFAs) acting as chromatin-active cofactors.

### 3.2. Microbial Taxa Drive Epigenetic Reprogramming: Remodeling of the Host Epigenome

The gut microbiome alters the epithelial epigenome via DNA methylation, histone modifications, reprogramming transcription without changing DNA sequence. Dysbiosis can amplify these deviations and steer pathways relevant to tumor initiation, progression, and clinical behavior. A central conduit is the SCFA pool—acetate, propionate, and butyrate—generated by microbial fermentation of dietary fiber and resistant starch. SCFAs serve as epithelial fuels and systemic messengers: they engage G-protein-coupled receptors on endocrine and immune cells and directly remodel chromatin by modulating histone acetyltransferases/deacetylases and influencing DNA demethylation machinery. In this capacity, SCFAs operate as bona fide epigenetic cofactors [41].

Butyrate exemplifies the metabolic–epigenetic coupling termed the “butyrate paradox.” In cancer cells that favor aerobic glycolysis (the Warburg effect), butyrate is inefficiently oxidized, accumulates in the nucleus, and acts as a histone deacetylase (HDAC) inhibitor, resulting in histone hyperacetylation and growth arrest. In contrast, normal colonocytes efficiently catabolize butyrate via β-oxidation and the TCA cycle, using it as a principal energy source that supports differentiation and barrier function. Contexts with defective DNA repair or mitochondrial metabolism can invert these outcomes: genetically unstable cells may exploit butyrate as an alternative fuel and, under selection, expand aggressive, angiogenic clones. In the healthy gut, SCFA production arises largely from *Firmicutes* (especially *Clostridiales*) and *Bacteroidetes*, with contributions from some *Actinobacteria* [41,42,43,44].

Beyond metabolites, specific taxa imprint epigenetic signatures. *F. nucleatum* has been associated with CIMP and MSI-high tumors, including promoter hypermethylation at genes such as *MLH1* [45,46,47]. The bacterium can also influence miRNA maturation: METTL3-dependent m6A processing enhances maturation of select miRNAs (e.g., miR-4717), which then repress tumor-suppressive targets like *MAP2K4*. Inflammatory signaling and oxidative stress driven by *F. nucleatum* increase reactive oxygen species and DNA double-strand breaks, perturbations that bias methylation patterns and stabilize oncogenic programs, and are linked to more aggressive, metastatic disease [48]. Complementing this, *E. coli* strains carrying the pks island produce colibactin, a genotoxin that alkylates adenine residues and induces DNA damage, leaving a characteristic mutational footprint. Genotoxic stress co-opts epigenetic control indirectly by stabilizing inflammatory transcriptional states and altering the balance of DNA and histone methylation/acetylation. At the tissue level, pks^+^ *E. coli* promotes an immunosuppressive, lipid-replete microenvironment that facilitates tumor progression and diminishes therapeutic responsiveness [49].

Across these layers, microbial cues—adhesins and toxins from *F. nucleatum* and ETBF, NF-Κb-activating signals from AIEC, and colibactin from pks^+^ *E. coli*—feed into shared host circuitry. Post-transcriptionally, miRNAs such as miR-21 (RASA1–MAPK), miR-31 (eIF4EBP1/2; STX12), miR-18a*/miR-4802 (ULK1/ATG7), and miR-1322 (CCL20) recalibrate autophagy, chemokines, and growth control. At the chromatin level, SCFAs and inflammation-linked signals shape histone acetylation/deacetylation and DNA methylation, reinforcing tumor-promoting states.

## 4. Population-Specific Risk: Situating Romania Within Lifestyle–Microbiome Evidence

At present, no Romania-specific CRC microbiome datasets are available. To avoid over-interpretation, we benchmark Romania against (i) large European CRC cohorts that integrate lifestyle exposures with microbiome features and (ii) Romanian metabolic cohorts (type 2 diabetes, metabolic syndrome) that report gut microbial and metabolite shifts relevant to CRC biology. Signals from metabolic cohorts are treated as hypothesis-generating rather than definitive CRC evidence. A consolidated summary of cohorts, assays, and key signatures is provided in Table 1.

### 4.1. European Evidence on Lifestyle–Microbiome Links and Implications for Romania

**Figure 1:**
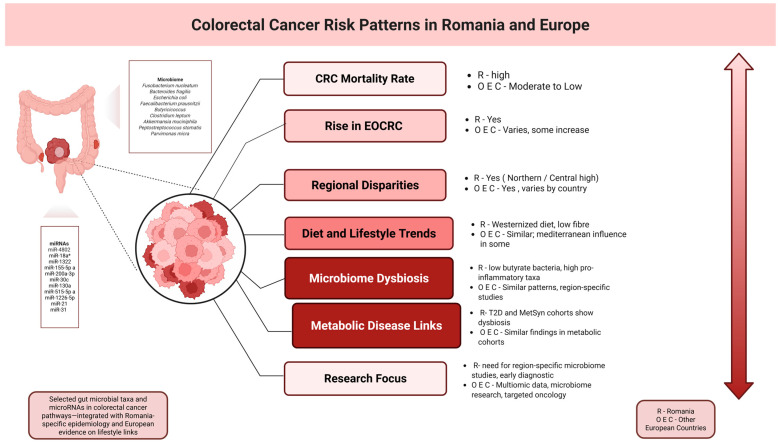
Lifestyle, microbiome, and CRC in Europe and Romania: rising incidence parallels obesity, alcohol, diet shifts, and sedentary habits; cohorts highlight regional disparities, and microbial markers. Created in BioRender. Simona, T. (2025). https://BioRender.com/sfheuqk (accessed on 9 May 2025).

Across Europe, combined healthy behaviors—healthy weight, physical activity, non-smoking, limited alcohol, and a prudent diet—associate with lower risk; in EPIC, each additional healthy factor reduced incidence, and the most favorable profile conferred ~one-third risk reduction [50]. Comparative analyses show that obesity, alcohol, and erosion of traditional diets can negate presumed protections; for example, Croatian data associate obesity and daily alcohol use with increased risk despite a Mediterranean backdrop [51]. Viewed through this lens (and as illustrated in Figure 1), Romania’s epidemiology mirrors continental trends: incidence is rising alongside Westernized eating (more red/processed meat and refined carbohydrates, less fiber and dietary diversity) and more sedentary routines. Spatial analyses indicate marked heterogeneity—highest incidence and mortality in the north and center, with lower figures in Bucharest and southern provinces [5].

Microbiome-anchored European cohorts sharpen the methodological and biological picture. In Belgium, a 589-patient colonoscopy cohort was partitioned into three diagnostic groups (no lesions, polyps, CRC) and profiled using quantitative microbiome profiling (QMP) 16S rRNA rather than relative profiling. By coupling absolute quantification with extensive metadata (including stool transit time, fecal calprotectin as an inflammation proxy, and BMI), the study reduced false positives/negatives and systematically probed covariates that can mask or create spurious taxon–disease associations. Contrary to much of the prior literature, well-known targets such as *Fusobacterium nucleatum* did not significantly associate with diagnostic groups once covariates were modeled. In contrast, associations for *Anaerococcus vaginalis*, *Dialister pneumosintes*, *Parvimonas micra*, *Peptostreptococcus anaerobius*, *Porphyromonas asaccharolytica*, and *Prevotella intermedia* remained robust, highlighting their future biomarker potential. The work also showed that colonoscopy “controls” (e.g., positive Fecal Immunochemical Test—FIT, but no lesions) can be enriched for the dysbiotic *Bacteroides2 enterotype*, complicating the definition of a truly healthy reference group. A large external validation cohort—among the best characterized at publication—underscored two design essentials for future studies: absolute quantification and rigorous covariate handling [52].

Metagenomic studies across France/Germany (with external validations) complement these findings. Fecal species-level classifiers distinguished cases from controls with performance comparable to fecal occult blood testing (FOBT) and improved further when combined with FOBT. Discriminative taxa included *Fusobacterium* spp., *Porphyromonas asaccharolytica*, and *Peptostreptococcus stomatis*. Functionally, CRC metagenomes showed greater utilization of host carbohydrates and amino acids and increased lipopolysaccharide metabolism, consistent with pro-inflammatory states. An Austrian metagenome-wide association analysis spanning healthy, advanced adenoma, and carcinoma identified microbial genes/strains/functions enriched by stage; risk-factor analysis indicated that high red-meat intake relative to fruits/vegetables associates with the outgrowth of bacteria that may create a more hostile gut milieu—findings that support fecal microbiome-based strategies for early diagnosis and intervention [53].

Taken together, European evidence delivers clear guidance for Romania: lifestyle aggregates materially shift risk; obesity and alcohol can outweigh nominal dietary protections; and, critically, study design matters—maybe using quantitative microbiome profiling (QMP) 16S rRNA rather than relative profiling, explicit capture of inflammatory and transit covariates, and careful control selection are prerequisites for robust, transportable biomarkers.

Because Romania lacks CRC-specific microbiome datasets, the most immediate window into local biology comes from metabolic cohorts that profile gut communities and metabolites. These do not establish CRC causality, but they can indicate whether Romanian exposures are tracking toward dysbiosis patterns repeatedly seen in CRC.

### 4.2. Romania’s Current Evidence Base, with Method Transparency and Hypothesis-Generating Inferences

Study-selection criteria (Romania and comparators). We selected studies that characterize the Romanian population in terms of gut microbiome composition and/or microbial-derived metabolites, preferably comparing healthy controls with patient groups whose conditions could plausibly be influenced by specific bacterial taxa. The same criterion guided the choice of international studies, with the caveat that most of those cohorts focused on colorectal cancer or colorectal precancerous lesions. As noted above, because no Romanian cohort with this pathology is currently available, we did not require a disease-specific inclusion criterion for Romania. A further criterion was methodological rigor: included studies had to use established approaches for profiling gut microbiome patterns—real-time PCR (qPCR)/NGS/16S rRNA gene sequencing—so that results are method-concordant with the broader literature. Two cohorts met these criteria—one in type 2 diabetes (T2D) and one in metabolic syndrome (MetSyn)—and are presented strictly as context for CRC, not as CRC evidence.

T2D cohort (n = 150; 105 T2D, 45 healthy). Findings: depletion of butyrate producers (*Faecalibacterium prausnitzii*, *Butyricicoccus*, *Subdoligranulum*, *Roseburia*), enrichment of Enterobacteriaceae and *Bacteroides*, and increases in *Dialister* and *Fusobacterium*; dysbiosis correlated with lipid parameters and blood pressure. *Akkermansia* was reduced [54].

Metabolic syndrome cohort (n = 60; 30 MetSyn, 30 controls). Findings: enrichment of Enterobacteriaceae, *Turicibacter*, and *Clostridium coccoides/leptum*; depletion of *Akkermansia muciniphila*, *F. prausnitzii*, and *Butyricicoccus*; lower butyrate with higher succinate/taurine; fungal shifts toward Saccharomyces/Aspergillus [55].

Interpretation and study design for Romania. Both Romanian cohorts align with motifs repeatedly observed in CRC elsewhere: loss of butyrate producers, gain of inflammation-prone/pathobiont taxa, and SCFA imbalance (↓ butyrate, ↑ succinate). These patterns are hypothesis-generating: they argue for Romanian CRC studies that (i) use QMP or metagenomics, (ii) record covariates with high confounding potential (fecal calprotectin, stool transit/moisture, BMI, medications), and (iii) define controls carefully, recognizing that screen-negative individuals may still exhibit dysbiotic enterotypes (e.g., *Bacteroides2*). Embedding standardized lifestyle measures (dietary diversity/fiber, alcohol, physical activity, anthropometry) will enable direct comparison to European benchmarks and reduce ambiguity between mechanistic and epidemiological inference.

European cohorts quantify how lifestyle aggregates and microbial community structure co-vary with risk, and they set methodological standards that minimize spurious associations. Romania’s rising burden—coupled with regional heterogeneity—fits this continental pattern. Local metabolic cohorts already indicate a drift toward CRC-relevant dysbiosis; a CRC-specific, method-transparent program that integrates diet, inflammation, microbiome, and metabolome is the logical next step to move from hypothesis generation to actionable evidence.

## 5. Beyond *Fusobacterium nucleatum*: Expanding Roles of Other Oncobacteria in Colorectal Cancer

Although *F. nucleatum* remains a principal microbial actor in CRC, additional taxa have direct, experimentally tractable effects on tumor biology. Two organisms, *Peptostreptococcus stomatis* and *Parvimonas micra*, understudied until now, also exhibit mechanisms through which microbial adhesion, host–receptor interactions, and immune modulation can drive tumor progression, shape prognosis, and impair targeted therapies.

### 5.1. Peptostreptococcus stomatis: ERBB2–MAPK Activation and Therapeutic Bypass

*P. stomatis* is enriched in colorectal tumors and adheres to and invades colonic epithelium, suppressing apoptosis and driving cell-cycle progression—phenotypes that increase tumor multiplicity and histologic grade in both genetic and inflammation-associated models. A surface adhesin, fructose-1,6-bisphosphate aldolase (FBA), engages epithelial integrin α6/β4 with micromolar affinity. This interaction is required for attachment, invasion, and downstream signaling: knockdown or antibody blockade of α6/β4 abrogates adhesion, blunts proliferation, and prevents pathway activation. *P. stomatis* elicits alternative activation of ERBB2 and propagates signaling through the MEK–ERK–p90RSK cascade, accompanied by increased integrin α6/β4 and phospho-ERBB2. In vivo, colonization compromises barrier proteins (E-cadherin, occludin), elevates high-grade dysplasia/adenocarcinoma, and augments colonic inflammation. Importantly, *P. stomatis*-driven ERBB2 signaling bypasses receptor tyrosine kinase inhibition: co-culture or intratumoral delivery diminishes the impact of EGFR inhibitors (cetuximab, erlotinib) in KRAS-wild-type settings and of BRAF blockade (vemurafenib) in BRAF^V600E tumors, whereas ERBB2 inhibitors (e.g., tucatinib, CP-724714) restore sensitivity. Overexpressing *P. stomatis* FBA or administering purified FBA reproduces adhesion, ERBB2–MAPK activation, and tumor-promoting effects, underscoring the sufficiency of the adhesin–integrin axis [56].

### 5.2. Parvimonas micra: Prognostic Significance, Th17 Polarization, and WNT Pathway Engagement

Across multiple cohorts, *P. micra* is consistently enriched in fecal and mucosal samples from patients versus controls; tumor tissue often shows higher abundance than adjacent normal mucosa. Elevated fecal *P. micra* independently predicts poorer 5-year survival after adjustment for age, sex, and stage. Experimentally, *P. micra* accelerates tumorigenesis in Apc^Min/+ mice, stimulates colonocyte proliferation in conventional animals, and remains pro-proliferative under germ-free monocolonization—indicating a direct epithelial effect. Conditioned media increase HT-29 viability and clonogenicity and activate WNT/β-catenin signaling). In vivo transcript profiling reveals induction of programs linked to proliferation (Mki67, Cdc20), invasion/metastasis (Snai1, Cdh2), stemness (Sirt1, Bmi1), and angiogenesis (Pgf, Angpt1, Flt1), alongside repression of apoptotic and DNA-damage response pathways. Immunologically, *P. micra* skews mucosal immunity toward a Th17 phenotype: colonic IL-17A, IL-22, and IL-23 increase, Th17 infiltration rises, and chemokine networks (including CCL20 and several CXCLs) are induced, reinforcing an inflammatory, tumor-supportive microenvironment. Notably, conditioned media from *P. micra* directly promote CD4^+^ T-cell differentiation toward IL-17^+^ cells, linking this bacterium to adaptive immune reprogramming that favors tumor progression [57].

Together, *P. stomatis* and *P. micra* extend the spectrum of tumor-associated microbes beyond *F. nucleatum* by acting through complementary axes. Their recurrent enrichment in patient cohorts, with causal evidence in mouse and organoid models, indicates that microbiome profiling can sharpen prognosis and guide microbiome-informed therapy design.

## 6. Conclusions

CRC increasingly appears as a systems disease in which the gut microbiome, host microRNAs, and epigenetic circuitry act together to shape tumor behavior. Convergent evidence shows that taxa such as *Fusobacterium nucleatum*, enterotoxigenic *Bacteroides fragilis*, and pks^+^ *Escherichia coli* engage NF-κB, WNT/β-catenin, and mTORC1 signaling while rewiring miRNA networks (miR-21, miR-31, miR-18a*/miR-4802, miR-1322), with downstream effects on inflammation, immune evasion, autophagy, metastatic fitness, and treatment resistance. These mechanistic links support the clinical development of microbial signatures integrated with circulating or tissue miRNAs as non-invasive biomarkers for earlier detection, refined prognostication, and prediction of therapeutic response. They also highlight actionable levers—nutritional and ecological modulation, blockade of microbial adhesins and toxins, and miRNA/epigenetic interventions—as rational complements to standard oncology.

Looking ahead, advancing from association to application will require longitudinal, covariate-controlled cohorts that reflect the epidemiologic and dietary realities of populations, including Romania, while validating microbial and epigenetic biomarkers for targeted prevention and therapy. Diet should be considered a modifiable component of care, not merely a background exposure; defining how specific dietary patterns during oncological treatment influence tolerance and efficacy is a priority. Trials of microbiome-directed therapeutics merit careful evaluation, including fecal microbiota transplantation (FMT), defined consortia, prebiotics/postbiotics, and SCFA-targeted strategies, with safety and scalability in view. A research agenda built around these principles can translate microbiome–miRNA biology into practical tools and interventions for CRC control in Romania and beyond.

## Figures and Tables

**Table 1:** Key gut microbiome findings across Romanian and other European cohorts.

Country/Study Context	Cohort	Assay	Gut Microbial Signature
Romania/T2D	n = 150 (105 T2D, 45 HC)	RT PCR/16S rRNA gene/SYBR Green primers Fecal samples	↓ Lactobacilli species, *Akkermansia muciniphila*, butyrate producers (*Faecalibacterium prausnitzii*, *Butyricicoccus*, *Subdoligranulum*); ↑ *Enterobacteriaceae*, *Fusobacterium*, *Dialister*
Romania/MetSyn	n = 60 (30 MetSyn, 30 HC)	RT PCR/16S rRNA gene/SYBR Green primers Fecal samples	↑ *Gamma Proteobacteria*, *Beta Proteobacteria*, *Enterobacteriaceae*, *Turicibacter*, *Clostridium* (*coccoides/leptum*); ↓ *Butyricicoccus sp.*, *Akkermansia muciniphila*, *Faecalibacterium prausnitzii*
Belgium/LCPM	n = 589 (referred for colonoscopy and colonic resections; 3 groups: CTLs; ADE; CRC) validation cohort	QMP Fecal samples	Significant differential abundance across groups: *Anaerococcus*, *Alistipes onderdonkii*, *Dialister pneumosintes*, *Fusobacterium nucleatum*, *Parvimonas micra*, *Peptostreptococcus anaerobius*, *Porphyromonas asaccharolytica*, *Prevotella intermedia*. Adjustment for calprotectin (±BMI, stool moisture) abolishes the absolute/relative *F. nucleatum*—CRC diagnosis association. Bact2 enterotype is overrepresented in colonoscopy patients, regardless of CRC status.
France/Germany	n= 156 population F(healthy/adenoma/CRC)/France n = 38 population G(CRC)/Germany n = 297 population H(healthy)/Germany; Denmark and Spain from other studies	RT PCR/16S rRNA Metagenomic shotgun sequencing Fecal and tissue samples	22-species signature: *Fusobacterium nucleatum* (subsp. *vincentii/animalis*), *Peptostreptococcus stomatis Porphyromonas asaccharolytica*, *Porphyromonas asaccharolytica*, *Clostridium hylemonae*, *Clostridium symbiosum* …—all enriched in CRC Early-stage CRC vs. controls: ↑ *Fusobacterium* spp.; ↑ *Peptostreptococcus stomatis* (strong enrichment) ↓ *Eubacterium rectale*, ↓ *Eubacterium eligens*, ↓ *Streptococcus* (negative association).
Austria	n = 156 (57 HC + 6 HC/AMS; 44 AA + 3 AA/AMS; 46 CRC)	Metagenomic shotgun sequencing Fecal samples	↑ *Actinomyces viscosus*, *Bifidobacterium animalis*, *Streptococcus thermophilus*, *Clostridium sp.*, *Streptococcus mutans*—HC ↑ *Streptococcus thermophilus* *Bifidobacterium animalis*, *Actinomyces viscosus*, *Bacteroides massiliensis*, *Paraprevotella clara* *Bacteroides dorei*—AA ↑ *Fusobacterium*, *Escherichia coli*, *Parvimonas*, *Bacteroides eggerthii*, *Sutterella wadsworthensis*, *Veillonella atypica*, *Bilophila*, *B.massiliensis*,*B.dorei*,*B.vulgates*, *Parabacteroides merdae*, *A.finegoldii*, *B.wadsworthia*, *Lachnospiraceae bacterium*, *Alistipes finegoldii*, *Bacteroides caccae*—*CRC*

Abbreviations: CRC—colorectal cancer; T2D—Type 2 Diabetes; MetSyn—Metabolic Syndrome; HC—healthy controls; HC/AMS—healthy controls/another manuscript samples (in line with the original article text); AA—advanced adenoma; AA/AMS—advanced adenoma/another manuscript samples (in line with the original article text); QMP—quantitative microbiome profiling; LCPM—Leuven CRC Progression Microbiome; CTLs—patients without evidence of colonic lesions; ADE—patients with polyps; Bact2—Bacteroides2 enterotype; RT-PCR—reverse transcription polymerase chain reaction; 16S rRNA—16S ribosomal RNA; ↓ indicates decreased abundance; ↑ indicates increased abundance.

## Data Availability

Not applicable.
